# Pressure cells for *in situ* neutron total scattering: time and real-space resolution during deuterium absorption

**DOI:** 10.1107/S1600576722010561

**Published:** 2022-12-01

**Authors:** Kazutaka Ikeda, Hidetoshi Ohshita, Toshiya Otomo, Kouji Sakaki, Hyunjeong Kim, Yumiko Nakamura, Akihiko Machida, Robert B. Von Dreele

**Affiliations:** a Institute of Materials Structure Science, High Energy Accelerator Research Organization (KEK), Tokai, Ibaraki 319-1106, Japan; b J-PARC Center, High Energy Accelerator Research Organization (KEK), Tokai, Ibaraki 319-1106, Japan; c School of High Energy Accelerator Science, Graduate University for Advanced Studies, Tsukuba, Ibaraki 305-0801, Japan; d Graduate School of Science and Engineering, Ibaraki University, Tokai, Ibaraki 319-1106, Japan; e National Institute of Advanced Industrial Science and Technology, Tsukuba, Ibaraki 305-8569 Japan; f Quantum Beam Science Research Directorate, National Institutes for Quantum and Radiological Science and Technology, Sayo, Hyogo 679-5148, Japan; g Advanced Photon Source, Argonne National Laboratory, 9700 South Cass Avenue, Argonne, IL 60439-4814, USA; Oak Ridge National Laboratory, USA; North Carolina State University, USA

**Keywords:** *in situ* neutron total scattering, pair distribution functions, hydrogen storage, reverse Monte Carlo methods

## Abstract

*In situ* gas-loading sample holders for two-dimensionally arranged detectors in time-of-flight neutron total scattering experiments have been developed to investigate atomic arrangements during deuterium absorption using time and real-space resolution. A single-crystal sapphire container was developed that allows conditions of 473 K and 10 MPa hydrogen gas pressure. High-resolution transient measurements detected deuterium absorption by palladium that proceeded within a few seconds. A double-layered container with thick- and thin-walled vanadium allowed conditions of 423 K and 10 MPa hydrogen gas pressure. The deuterium occupation sites of a lanthanum–nickel–aluminium alloy are discussed in detail on the basis of real-space high-resolution data obtained from *in situ* neutron scattering measurements and reverse Monte Carlo structural modeling.

## Abstract 2

*In situ* gas-loading sample holders for two-dimensionally arranged detectors in time-of-flight neutron total scattering experiments have been developed for the structural analysis of the deuterium absorption process using time and real-space resolution.

## 1. Introduction

Hydrogen, an abundant resource with a low environmental impact, is attracting attention as an energy carrier, and safe and efficient hydrogen storage technology is an important issue for a future hydrogen energy society. Major scientific interest has been drawn to the development of hydrogen storage materials, such as metal/alloy hydrides, complex hydrides, ammonia borane and metal–organic frameworks, which must meet the requirements of high storage capacity, suitable thermodynamic properties, high reversible durability, and high absorption and desorption rates.

To understand these properties and develop applicable materials, it is important to clarify the processes of hydrogen absorption and desorption from the viewpoint of atomic arrangement. To date, dislocations and lattice strains have been investigated by analyzing the average and local structures of metal lattices, primarily by *in situ* X-ray diffraction measurements during hydrogen absorption and desorption (Sakaki *et al.*, 2018 ▸; Lin *et al.*, 2020 ▸; Zhang *et al.*, 2022 ▸). The location of the hydrogen in these materials is the most important information required to understand and develop materials with these requirements (Hansen & Kohlmann, 2014 ▸; Lin *et al.*, 2020 ▸; Zhang *et al.*, 2022 ▸).

Taking advantage of the direct interaction of neutrons with hydrogen, the location of hydrogen (deuterium) in various hydrogen storage materials has been determined by *in situ* elastic neutron scattering. However, many metallic elements used as container materials react with hydrogen at high temperatures and pressures, leading to embrittlement, which is a limitation for the *in situ* measurement of chemical reactions. The sample container material must be stable under experimental gas pressure and temperature conditions, be chemically inert to hydrogen, have low absorption and background neutron scattering intensity, and have a removable diffraction signal. High-strength materials, such as Inconel, have been considered to overcome hydrogen embrittlement, which naturally introduces high diffraction peaks (Remhof *et al.*, 2009 ▸). Although single-crystal sapphire vessels have been developed and structural changes during hydrogen absorption have been investigated on a minute-by-minute basis, detectors placed at limited scattering angles in a constant-wavelength system for the angular dispersion method sometimes produce sapphire diffraction peaks that obscure the sample’s diffraction peaks, even with carefully chosen installation orientations (Kohlmann *et al.*, 2009 ▸; Widenmeyer *et al.*, 2013 ▸; Finger, Hansen & Kohlmann, 2021 ▸; Finger, Kurtzemann *et al.*, 2021 ▸; Finger *et al.*, 2022 ▸). In contrast, no attempt has been made to use a single-crystal sapphire container for detectors arranged in two dimensions in the time-of-flight method (*i.e.* an accelerator-driven neutron diffractometer in which short-wavelength neutrons are effective) to obtain a whole diffraction pattern with no missing sections, even if the Bragg peaks are shifted during hydrogen absorption and desorption reactions.

In this study, we apply a single-crystal sapphire container to the two-dimensionally arranged detectors in time-of-flight *in situ* neutron scattering experiments to derive diffraction patterns for Rietveld refinement and demonstrate that it is possible to investigate seconds-long structural changes during the hydrogen absorption process. For other materials, experiments using the time-of-flight method with BN shielding in an Inconel-lined neutron elastic zero scattering Zr_2.1_Ti vessel (Gray *et al.*, 2007 ▸), Cd shielding in a stainless steel vessel (Nakamura *et al.*, 2009 ▸) and a Cu-coated vanadium vessel (Iwase *et al.*, 2011 ▸) and low-temperature measurements in a pure vanadium vessel (Sakaki *et al.*, 2013 ▸) have been performed. However, the scattering angle or temperature range is limited, making it difficult to reach the high-*Q* region necessary for high resolution in real space. We have also developed a double-walled vanadium container and carried out a local structural analysis of LaNi_5_-based alloy deuterides by deriving a pair distribution function (PDF) with high resolution in real space while considering safety issues. A detailed investigation of localized short-range atomic arrangements for LaNi_4.5_Al_0.5_ deuteride using reverse Monte Carlo (RMC) structural modeling (McGreevy & Pusztai, 1988 ▸) from neutron total scattering measurements has also been undertaken.

Structural analysis of hydrogen storage alloys requires the analysis of disordered atomic arrangements of hydrogen occupying interstitial sites in the crystal structure of ordered arranged metal lattices. This study examines the performance of two types of *in situ* gas-loading sample containers with different chemical stability for hydrogen gas and real-space resolution associated with data processing methods, and then demonstrates their usefulness from the results of averaged and local structure analyses.

## 2. Experimental procedures

### 2.1. Neutron total scattering measurements

Neutron total scattering experiments were undertaken using the NOVA neutron total scattering instrument (BL21 beamline) with a decoupled liquid hydrogen moderator, an incident flight path of 15 m and a scattered flight path of 1.2–1.3 m at the 90° detector bank [0.08 < *d* < 6.3 Å; *d* = 2π/*Q* = λ/(2sinθ)] connected to the 400 kW spallation neutron source at the Japan Proton Accelerator Research Complex. Contamination from the background intensities of the sample container and the instrument was subtracted, and the neutron attenuation factors of the sample and sample container were calibrated. Structural refinement of the diffraction data in reciprocal space and real space was carried out by Rietveld refinement using the computer program *Z-Rietveld* (Oishi *et al.*, 2009 ▸; Oishi-Tomiyasu, 2012 ▸) and PDF refinement using *PDFgui* (Farrow *et al.*, 2007 ▸), respectively. Si powder (NIST SRM 640d) was used as a standard to determine the relationship between lattice spacing *d* and time of flight, and to evaluate the accuracy of the crystal and local structural analyses. Palladium powder was purchased from Kojima Chemicals Co. Ltd. NaAlD_4_–TiCl_3_ was synthesized as described in a previous study (Ikeda *et al.*, 2021 ▸). LaNi_4.5_Al_0.5_ was provided by Nippon Denko Co. Ltd and prepared as described in a previous study (Sakaki *et al.*, 2009 ▸). Crystal and local structure analyses were performed using sapphire, which is chemically stable but contaminated with diffraction signals that should be excluded, and vanadium, which requires caution in high-temperature experiments in a hydrogen atmosphere.

### 2.2. Reverse Monte Carlo modeling

The atomic arrangements of LaNi_4.5_Al_0.5_ before and after hydrogen absorption were investigated by fitting to the diffraction pattern and its Fourier transform as a PDF using *GSAS-II* (Toby & Von Dreele, 2013 ▸) in conjunction with *RMCProfile* (Tucker *et al.*, 2007 ▸). The method used 16 × 16 × 20 superlattices (La_5120_Ni_23173_Al_2427_ in 80.7 × 80.7 × 80.5 Å^3^ and La_5131_Ni_23207_Al_2419_D_27108_ in 85.6 × 85.6 × 85.4 Å^3^) for the RMC structural modeling using diffraction patterns and a PDF of 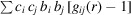 [*c*_*i*_ and *b*_*i*_ are the concentration and coherent bound scattering length, respectively, of atom *i*, and *g*_*ij*_(*r*) is the partial pair distribution function of atoms *i* and *j*, respectively]. Initial structures were created in which each atom was randomly placed to match the Rietveld refinement results described above and reproduced by modeling. During the modeling, translation, deuterium and vacancy swaps, and Ni and Al swaps were attempted with probabilities of 0.6, 0.2 and 0.2, respectively. The maximum translation distances were restricted to 0.10 Å for deuterium and 0.05 Å for the metals. The atoms were constrained to move no closer together than the predefined cut-off distances of 2.20 Å in LaNi_4.53_Al_0.47_ and 1.30 Å in LaNi_4.53_Al_0.47_D_5.27_.

## 3. Results

### 3.1. Sapphire container

**Figure 1:**
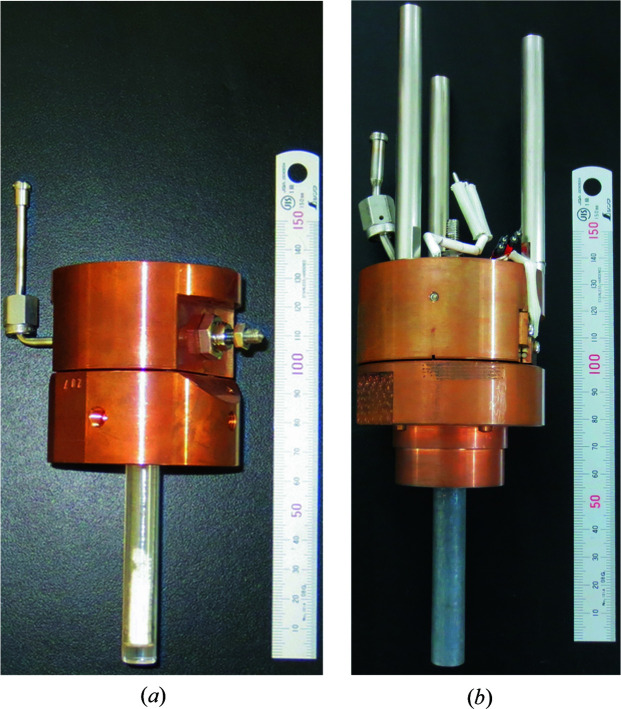
Setups for (*a*) the single-crystal sapphire sample container with powder sample (NaAlD_4_–0.02TiCl_3_) and (*b*) the vanadium sample container for the *in situ* neutron total scattering experiments.

A single-crystal sapphire container (Orbe Pioneer Ltd) with an inner diameter of 5.8 mm, a wall thickness of 2.5 mm and a base thickness of 5.0 mm was fabricated to meet the study’s requirements for safety and *in situ* neutron diffraction measurements [Fig. 1 ▸(*a*)]. First, the quality of the single-crystal sapphire container was investigated by neutron diffraction. NOVA is a neutron total scattering instrument that uses the time-of-flight method and consists of five position-sensitive detector (PSD) banks. As shown in Fig. S1(*a*) in the supporting information, the Laue pattern detected in the 90° bank confirmed that the single crystal was of good quality: the Laue spots were only detected at wavelength and scattering angle conditions that satisfied the Bragg equation. Since single crystals have a significantly higher Bragg peak intensity at specific detection pixels of the two-dimensionally arranged PSD, the diffraction signals (coherent scattering components) of such single crystals alone can be removed by setting an upper threshold value. In contrast, the diffraction signal of the powder inserted into the single-crystal container is uniformly distributed across the detector [Fig. S1(*b*)] and therefore a powder diffraction pattern is obtained from the region from which the single-crystal signals have been excluded.

**Figure 2:**
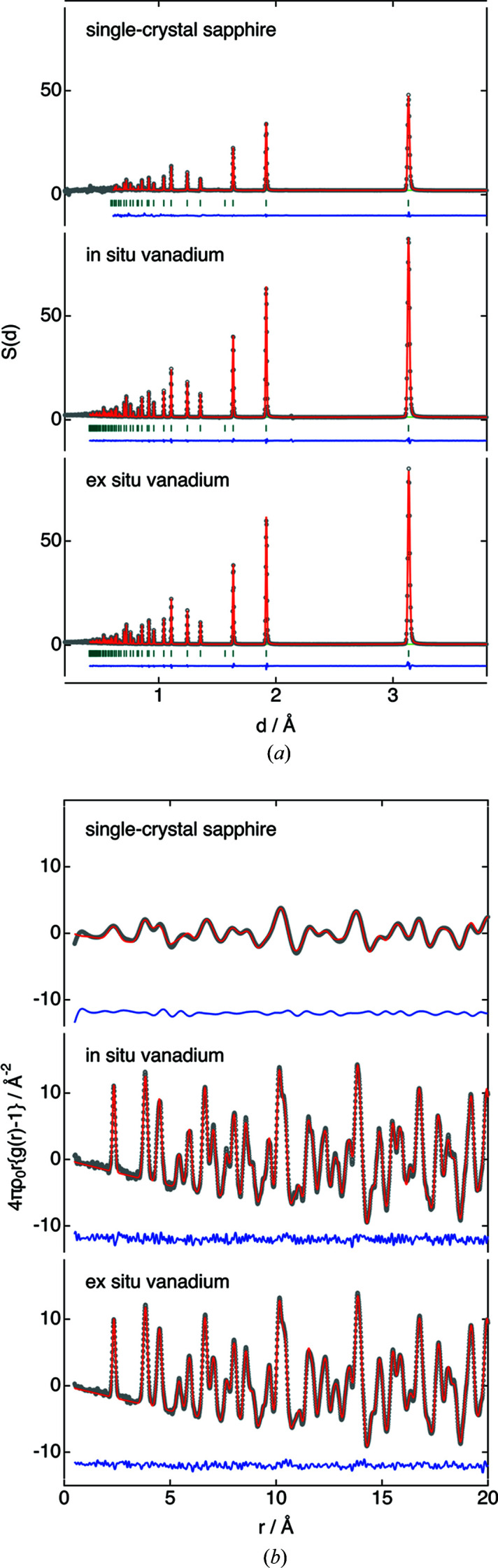
(*a*) Neutron diffraction patterns and Rietveld refinements, and (*b*) the PDF refinements for Si powder sealed in the single-crystal sapphire, *in situ* vanadium and *ex situ* vanadium sample containers. Rietveld refinement results: observed (circles), calculated (line) and residual (line below the vertical bars) diffraction profiles. PDF refinement results: observed (circles), calculated (line) and residual PDF profiles.

We performed neutron powder diffraction measurements on Si to compare the quality of the data obtained from the single-crystal sapphire container and an *ex situ* atmospheric-pressure vanadium container (with an inner diameter of 5.8 mm and a wall thickness of 0.1 mm) [Fig. 2 ▸(*a*)]. Data for the Si powder inserted into the single-crystal sapphire container and the empty container were obtained by the method described above. The 001 axis of the single crystal grew along the axis of the cylinder, but the crystal axis was not important. As shown in Fig. S1, the two containers had different crystallographic axes in the radial direction, but the total scattering curve structure factor *S*(*Q*) was obtained by typical data reduction, such as absorption correction and subtraction. However, this data processing method makes it difficult to distinguish between diffraction signals originating from the single-crystal sapphire and the Si powder in the high-*Q* region; therefore, an averaged crystal structure refinement was performed over a lattice spacing range of 0.6–4.0 Å (Table S1).

The FWHMs of the Si 111 diffraction peak from Gaussian fitting were 0.1805 (7) and 0.1802 (7) Å, respectively, for the single-crystal sapphire and *ex situ* vanadium containers. Since the design resolution of the NOVA 90° bank is approximately 0.5% (FWHM 0.016 Å), the peak widths derived from the sample could be evaluated. The atomic displacement parameter for the data from the single-crystal sapphire container was slightly larger than that for the *ex situ* vanadium container, but the reliability factors were comparable. Another advantage of two-dimensionally arranged detectors for the time-of-flight method is that the lattice spacing can be detected over a wide range of scattering angles, which produces a diffraction pattern with no missing signals. In contrast, for detectors placed at limited scattering angles in one dimension, the removal of single-crystal signals leads to discontinuities in the diffraction curve.

Before the experiment under hydrogen gas pressure, a pressure resistance test was conducted for the single-crystal sapphire container. No pressure drop was detected at 40 MPa of air at room temperature. The container flange was connected to the high-pressure gas piping through a copper holder (Pretech Co. Ltd) that could be sealed with a polyether ether ketone resin ring, and a vanadium radiation shield was placed around the container. The top surface of the holder was thermally connected to a copper component with an embedded cartridge heater (50 W) and a Gifford–McMahon refrigerator, allowing *in situ* neutron scattering experiments at temperatures up to 473 K and hydrogen/deuterium gas pressures up to 10 MPa. The copper holder had a built-in valve, and by connecting the sealed sample container in a glove box, drawing a vacuum inside the piping and then opening the valve, the sample could be used for the experiments without exposure to air.

**Figure 3:**
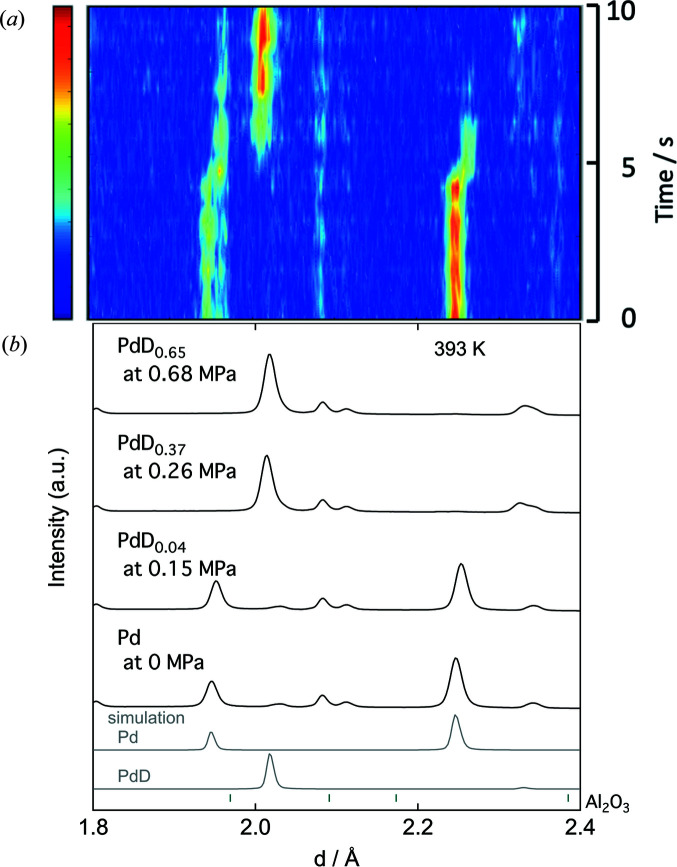
(*a*) Time transient (1 s intervals) and (*b*) static neutron diffraction patterns of palladium powder sealed in the single-crystal sapphire container.

Next, to confirm the validity of this container for *in situ* experiments, the deuterium absorption process of palladium was measured by neutron diffraction. Palladium inserted into the single-crystal sapphire container was maintained at 393 K, and deuterium was introduced stepwise up to 0.7 MPa to form a solid-solution phase followed by a hydride phase. A deuterium solid-solution phase (α-PdD_0.04_) with a lattice expansion of 0.3% relative to metallic palladium was formed at an equilibrium pressure of 0.15 MPa, and a diffraction pattern of the deuteride phase (β-PdD_0.65_) with periodically arranged deuterium atoms was reversibly obtained at 0.68 MPa [Fig. 3 ▸(*b*)] in conjunction with the measurement of the deuterium pressure–composition (*p*–*c*) isotherms (Fig. S2) (Flanagan & Oates, 1991 ▸). Data were obtained at 1 s intervals after introducing the deuterium gas all at once, which eventually reached an equilibrium pressure of 1 MPa to complete the deuterium absorption reaction of palladium within 10 s [Fig. 3 ▸(*a*)]. The structural change from metallic palladium to the deuteride phase via the deuterium solid-solution phase could be detected within seconds. These diffraction patterns appropriately excluded Al_2_O_3_ diffraction peaks originating from the sapphire. Although the diffraction peaks of the thermocouple were included around *d* = 2.1 Å in the current data, a diffraction pattern without any thermocouple impurity peaks can be obtained by controlling the sample temperature using a calibration curve between the set temperature and the sample section temperature of the copper holder. Structural analysis was also performed with LaMg_2_Ni–D (Sato *et al.*, 2017 ▸).

PDF data can also be obtained by Fourier transformation of the total scattering curve [Fig. 2 ▸(*b*)]. Since the real-space resolution of the PDF is proportional to the inverse of *Q*_max_ (Nield & Keen, 2001 ▸; Egami & Billinge, 2003 ▸), and the single-crystal sapphire container was limited to about *Q* = 10 Å^−1^, the standard deviations of the lattice constants and atomic displacements were relatively large (Table S1). Furthermore, second-by-second measurements may still be insufficient to obtain PDF data with sufficient statistical accuracy. Nevertheless, it is shown for the first time that chemically rigid single-crystal sapphire containers are effective for hydrogen gas experiments, and the PDF can also be derived.

### 3.2. *In situ* vanadium container

To compensate for the limited real-space resolution of the PDF measured by the single-crystal sapphire container, other materials were considered. Thin-walled vanadium-based containers with low coherent scattering lengths are often used for neutron diffraction measurements. Therefore, we designed a thick-walled *in situ* vanadium container with an inner diameter of 8.0 mm, a wall thickness of 1.5 mm and a bottom thickness of 4.0 mm to provide a pressure resistance of 40 MPa. Vanadium is inherently embrittled when reacted with hydrogen to form hydrides, but the hydrogen absorption reaction proceeds at around room temperature only after being baked in a vacuum above 473 K (Fig. S3). This is presumably due to the passivation of the surface oxide layer, so the surface of the container was intentionally oxidized by holding it at 773 K for 12 h in air. The powder sample was inserted into an open and thin-walled *ex situ* vanadium container and then sealed into the thick-walled *in situ* container to form a double structure. A typical hydrogen storage metal/alloy will expand in volume by about 30% due to hydrogen absorption, generating tens of megapascals of stress on the inner walls of the vessel (Okumura *et al.*, 2021 ▸). Therefore, sample-derived stresses must be considered in addition to the high-pressure gas. If the inner *ex situ* vessel fails due to volume expansion caused by the sample absorbing hydrogen, the vanadium in the newborn surface will instantly react with hydrogen/deuterium gas (diffraction peaks of VD_0.5_ or VD_2_ will be detected), thus avoiding damage (gas leakage) to the outer container. The thick-walled *in situ* vanadium container was mounted in a valved copper holder (Pretech Co. Ltd) with an embedded cartridge heater (50 W) to provide the setup for *in situ* neutron scattering experiments at temperatures up to 423 K and hydrogen/deuterium gas pressures up to 10 MPa [Fig. 1 ▸(*b*)].

Next, we evaluated the diffraction pattern and the PDF of Si powder inserted into the *in situ* vanadium container by neutron total scattering measurements (Fig. 2 ▸). Compared with the *ex situ* containers, the vanadium thickness had little effect on the diffraction pattern and the PDF was observed. To avoid problems measuring at high temperatures, the *in situ* neutron total scattering pattern (Fig. S4) and *P–C–T* curve of NaAlD_4_–TiCl_3_ up to 10 MPa at 403 K were measured simultaneously (Ikeda *et al.*, 2021 ▸). NaAlD_4_, identified at room temperature after synthesis, decomposed to NaD and Al by desorbing deuterium under a vacuum at 403 K. Later, when the deuterium pressure was increased, the NaAlD_4_ was reversibly combined via Na_3_AlD_6_. Equilibrium determination when measuring this system’s *P–C–T* curve took about 3 h, but there was no leakage of high-pressure gas over several days of step-by-step airtightness. The *Q* region equivalent to that of the *ex situ* vanadium is available (Table S1), and other measurements have already been made for Y_0.8_Mg_1.2_Ni_4_D_3.4_ and Y_1.1_Mg_0.9_Ni_4_D_3.9_ at 5 MPa D_2_ and 323 K (Sato *et al.*, 2020 ▸).

### 3.3. *In situ* neutron total scattering experiments of LaNi_4.5_Al_0.5_

Since the thick-walled *in situ* vanadium container could be used up to the high-*Q* region and was expected to provide high resolution in real space in the PDF data, a detailed structural analysis of *AB*_5_ alloy deuterides was carried out. *AB*_5_ alloy hydrides, typically LaNi_5_, have long been known as hydrogen storage alloys, and the results of many crystal structure analyses have been reported (Fischer *et al.*, 1977 ▸; Percheron-Guégan *et al.*, 1980 ▸; Yartys *et al.*, 1982 ▸; Thompson *et al.*, 1986 ▸; Lartigue *et al.*, 1987 ▸; Latroche *et al.*, 2004 ▸; Joubert *et al.*, 2021 ▸). The symmetry is hexagonal, with the hydrogen occupying the *O* (*A*2*B*4) and *T* sites (*A*2*B*2, *AB*3, *B*4). Metal *B* sites are substituted with aluminium or other metals to control the hydrogen storage and charge–discharge characteristics of the Ni–*M*H battery’s negative electrode (Achard *et al.*, 1982 ▸; Crowder *et al.*, 1982 ▸; Westlake, 1983*b* ▸; Percheron-Guégan *et al.*, 1985 ▸; Latroche *et al.*, 1992 ▸; Du *et al.*, 2003 ▸; Nakamura *et al.*, 2004 ▸). On the basis of abundant information on the crystal structure of *AB*_5_ alloy hydrides and on the Switendick criterion (Switendick, 1979 ▸) and the Westlake empirical rule (Westlake, 1983*a* ▸; Rao & Jena, 1985 ▸), the H—H shortest distance and the minimum space have been deduced, and the hydrogen occupation sites have been analyzed. However, since no PDF has been studied for the *AB*_5_ series of crystalline hydrides, *in situ* neutron total scattering measurements were performed to investigate in detail the atomic arrangement of the metal and hydrogen atoms.

**Figure 4:**
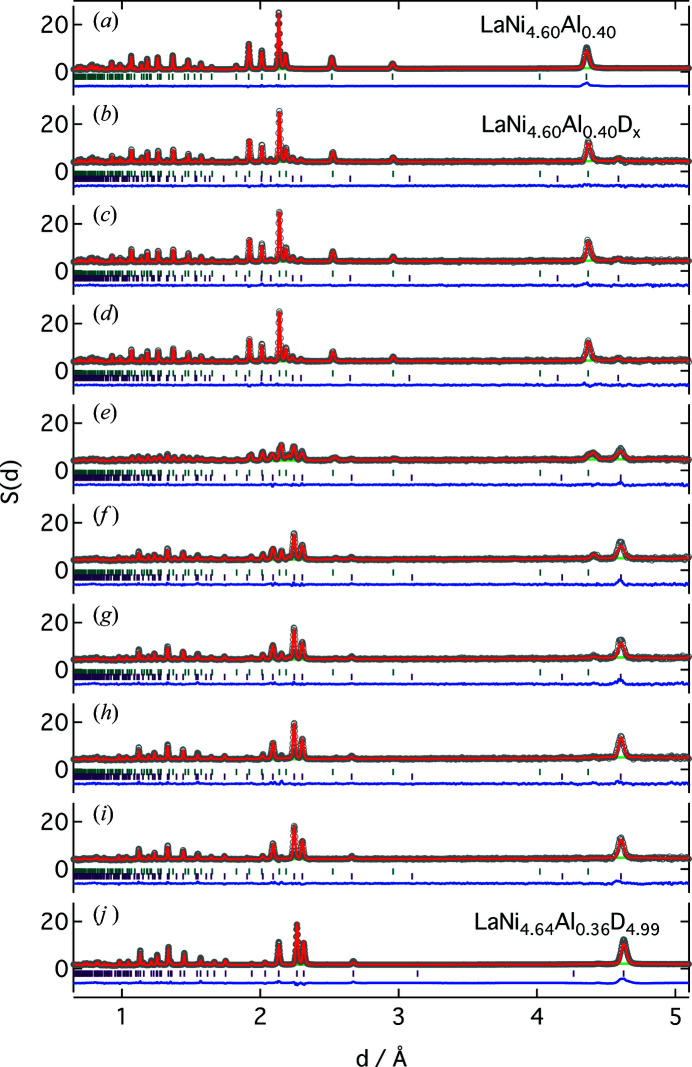
Neutron diffraction patterns and Rietveld refinements for LaNi_4.5_Al_0.5_ (*a*) before, (*b*)–(*i*) during (10 s intervals) and (*j*) after deuterium absorption at room temperature. Rietveld refinement results: observed (circles), calculated (line) and residual (line below the vertical bars) diffraction profiles. Bragg reflection positions are from LaNi_4.60_Al_0.40_ and LaNi_4.64_Al_0.36_D_4.99_.

The results of the Rietveld refinement in space group *P*6/*mmm* (No. 191) for the diffraction patterns of LaNi_4.5_Al_0.5_ before and after deuterium absorption at room temperature are shown in Fig. 4 ▸ and Table S2. The Rietveld refinement before deuterium absorption shows that this structural model works well, and the crystal structure information for a single phase of LaNi_4.6_Al_0.4_ and lattice constants of *a* = 5.042911 (9) Å and *c* = 4.027995 (11) Å were obtained. After deuterium absorption up to an equilibrium pressure of 1.8 MPa at room temperature, the lattice spacings lengthened [to *a* = 5.354025 (15) Å and *c* = 4.274487 (18) Å] and no new peaks appeared. The lattice is expanded by about 20% due to deuterium absorption, but no superlattice diffraction was detected and the single phase was maintained. These results are consistent with previously reported results (Percheron-Guégan *et al.*, 1980 ▸).

The Rietveld refinements show that the atomic displacement factors of La and Ni/Al are increased slightly and significantly, respectively, by deuterium absorption [*U*_La_ 0.00613 (6) Å^2^ → 0.00786 (7) Å^2^ and *U*_Ni/Al_ 0.00438 (4) Å^2^ → 0.01545 (4) Å^2^]. These results suggest that the atomic positions have deviated from their average positions upon deuterium absorption. The atomic displacements of deuterium obtained from the Rietveld refinement were quite large; therefore, the deuterium occupation of the 6*i* site (*A*2*B*4 site in the *AB*_5_ system) at (1/2, 0, 0.11996 (6)) could be split into the 12*n* site at (0.471 (3), 0, 0.103 (1)). The two models with deuterium on the 6*i* and 12*n* sites were compared, but the reliability factor improved by only 0.02%; therefore, the model with the 6*i* sites of higher symmetry was chosen for this study.

The transition process from the deuterium solid-solution phase to the deuteride phase up to an equilibrium pressure of 1.8 MPa at room temperature was investigated every 10 s, but a metastable intermediate phase was not detected (Fig. 4 ▸) (Joubert *et al.*, 2006 ▸; Gray *et al.*, 2011 ▸; Mohammadshahi *et al.*, 2017 ▸). The lattice constant of the intermediate phase is close to that of the hydride (Machida *et al.*, 2014 ▸) and it is difficult to distinguish between them in neutron diffraction, where the main scattering factor is the diffraction peak of deuterium, whose atomic displacement is larger than that of the metal. Additionally, the low fraction of the intermediate phase formed in aluminium-substituted LaNi_5_ may be the reason why it was not detected (Nakamura & Akiba, 2000 ▸).

**Figure 5:**
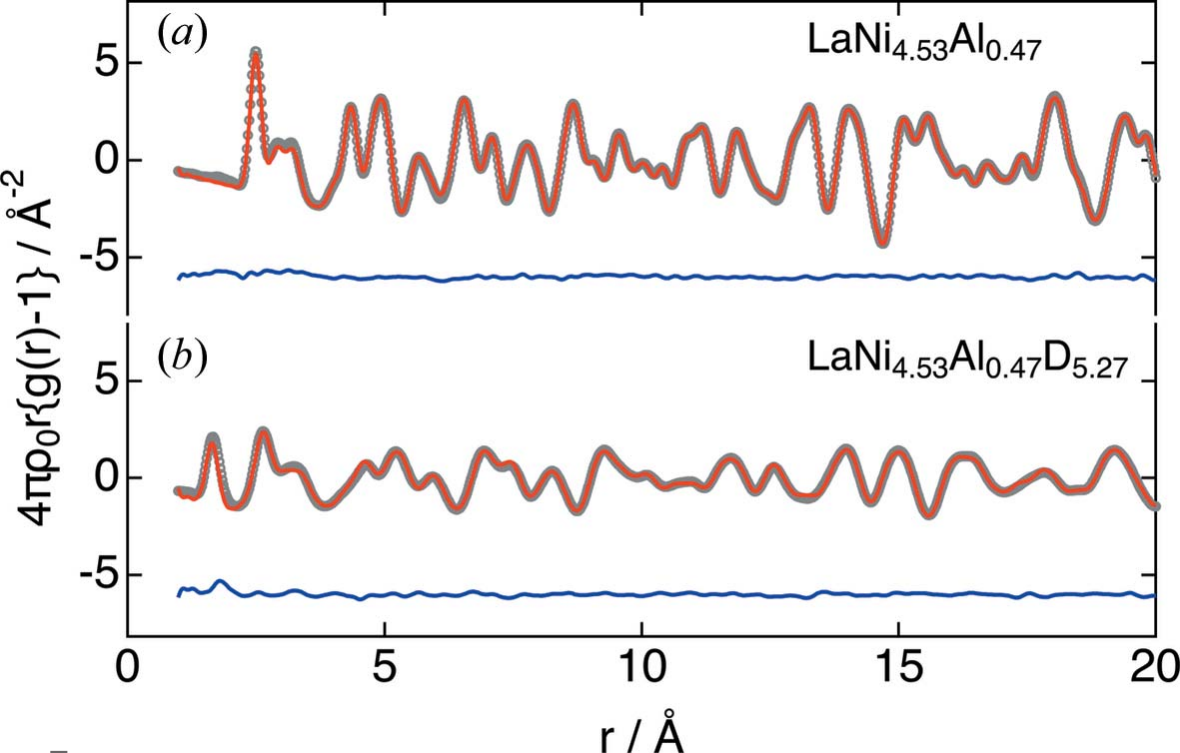
PDF refinements of LaNi_4.5_Al_0.5_ (*a*) before and (*b*) after deuterium absorption at room temperature. PDF refinement results: observed (circles), calculated (line) and residual PDF profiles.

The refinement results with the initial structure of the Rietveld refinement parameters for the PDF for LaNi_4.5_Al_0.5_ before and after hydrogen absorption are shown in Fig. 5 ▸ and Table S2. The PDF patterns have changed drastically with deuterium absorption, indicating that deuterium with its large neutron scattering length has dissolved in the interstitial structure. The lattice constants increased from *a* = 5.04210 (2) Å and *c* = 4.02472 (4) Å to *a* = 5.35283 (5) Å and *c* = 4.27084 (7) Å. Similarly to the Rietveld results, the atomic displacements of the metal elements were increased by deuterium absorption [*U*_La_ 0.013 (1) Å^2^ → 0.014 (1) Å^2^, *U*_Ni1_ 0.013 (1) Å^2^ → 0.021 (1) Å^2^ and *U*_Ni2/Al2_ 0.010 (1) Å^2^ → 0.016 (1) Å^2^], suggesting that the metal atom positions have deviated from their average positions.

The diffuse scattering component in *S*(*Q*) was properly reduced, resulting in a slight change in composition relative to the Rietveld refinement, which improved the accuracy of the subsequent analysis. A partial PDF simulation pattern was then evaluated on the basis of the refined crystal structure (Fig. S5). Since the PDF refinement uses an averaged structure based on the crystal structure information of the unit cell, there were D–D correlations even at *r* < 1 Å due to simultaneous deuterium occupation with low occupancy. In other words, the slightly insufficient refinement of LaNi_4.53_Al_0.47_D_5.27_ at *r* < 2.1 Å was due to the inclusion of D–D correlations that were not present, in addition to D–Ni. The function used for the PDF refinement was [*g*(*r*) − 1] multiplied by *r*, which emphasizes the structural information in the medium range and de-emphasizes the short-range region. However, *g*(*r*) accurately extracted information in the short range, allowing for a detailed discussion of the intrinsic local structure around the deuterium.

For this situation, RMC structural modeling of 16 × 16 × 20 superlattices (La_5120_Ni_23173_Al_2427_ in 80.7 × 80.7 × 80.5 Å^3^ and La_5131_Ni_23207_Al_2419_D_27108_ in 85.6 × 85.6 × 85.4 Å^3^) was performed to investigate the detailed local structure (Fig. S6). Although the atomic arrangement is finite, unlike an ideal crystal, the model was constructed to reproduce the measured information in reciprocal and real space. Note that to separate the first (D–Ni/Al) and second (D–La, Ni–Ni and Ni–Al) pair correlation peaks of LaNi_4.53_Al_0.47_D_5.27_, a Fourier transform using *S*(*Q*) up to *Q*_max_ = 50 Å^−1^ was required, as shown in Fig. S7, and *in situ* neutron total scattering experiments using double-walled vanadium containers can provide such data.

**Figure 6:**
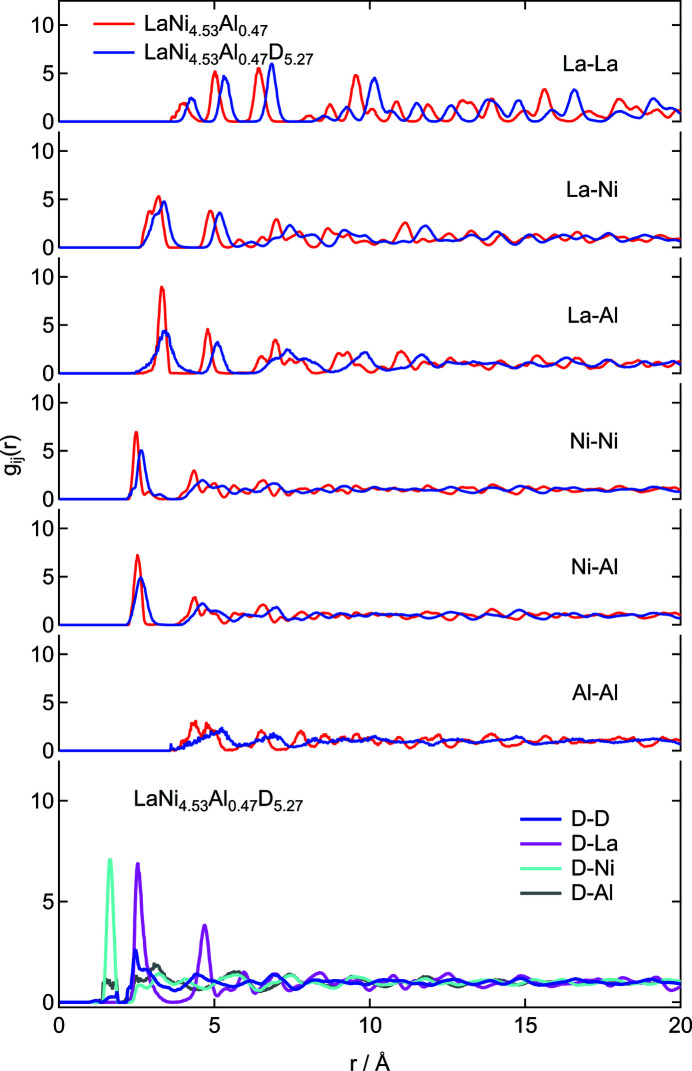
Partial PDF of LaNi_4.5_Al_0.5_ before and after deuterium absorption at room temperature.

The partial correlations obtained from the modeling are shown in Fig. 6 ▸. Both metal–metal correlations are elongated and have broadened distributions due to deuterium absorption, which is consistent with the Rietveld and PDF refinement results. The initial structure, with about 12% of the 3*g* site Ni partially substituted for Al in the Rietveld refinement, shows that Al–Al correlations appear at the same distance (*r* ≃ 2.5 Å) as the Ni–Ni correlations with composition-dependent intensity in the averaged structure. However, by swapping Ni for Al in the random configuration as the initial structure, no possible first nearest Al–Al correlation at the 3*g* site appears. Such correlations, which only appear at *r* > 3.5 Å after the modeling, could properly characterize the atomic arrangement. The model with crystal structure information for the metal–metal correlations approaching *r* < 1 Å in the LaNi_5_-based system was rejected because the pair correlation had not been measured. Similarly, the D–D correlations expressed in the PDF refinement by the average structural model for *r* < 1 Å were almost non-existent in the RMC modeling results, confirming the Switendick criteria and the Westlake empirical rule that D–D cannot be closer than 2 Å (Switendick, 1979 ▸; Westlake, 1983*a* ▸), as shown in the experimental results. While an average structural analysis can only guess the nearest-neighbor distance from the sites occupied by atoms and their occupancies, the local structure analysis presented in the current study allows experimental conclusions to be drawn.

**Figure 7:**
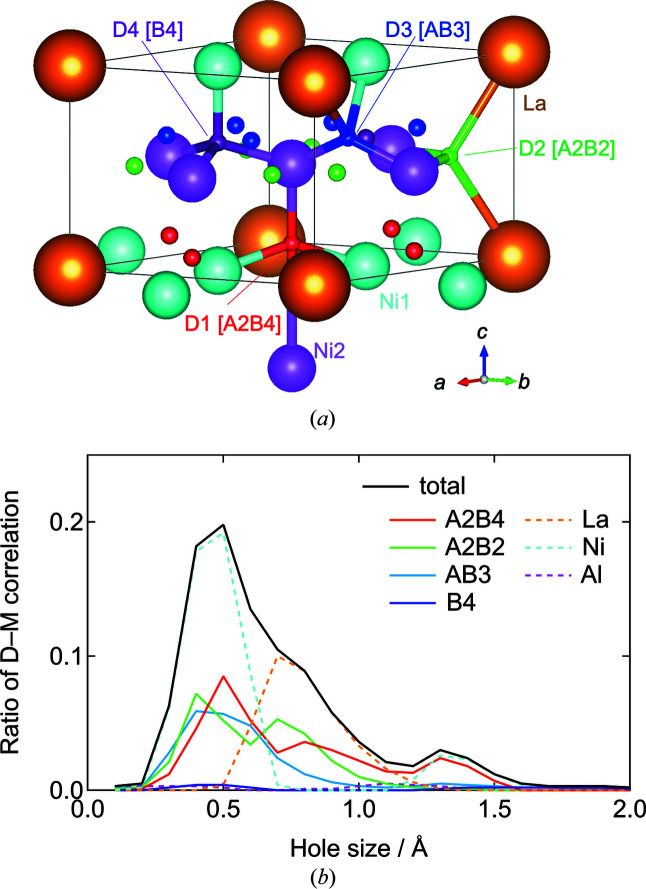
(*a*) Crystal structure with space group *P*6/*mmm* (No. 191) and (*b*) hole-size distribution of the deuterium occupation sites *A*2*B*4, *A*2*B*2, *AB*3 and *B*4 for LaNi_4.5_Al_0.5_ after deuterium absorption, indicated using the ratio of each D–*M* correlation to the total. The distribution using the ratio to constituent elements is also shown.

The local structure around hydrogen, which is directly related to hydrogen storage properties, is characterized by the first nearest pair correlations of D–Ni and D–Al (*r* ≃ 1.7 Å) and D–La (*r* ≃ 2.5 Å). From these atomic coordinates and the atomic radii of Ni (1.246 Å), Al (1.432 Å) and La (1.877 Å) (Westlake, 1983*b* ▸), the space occupied by deuterium was further analyzed in detail. In the atomic arrangement after modeling, the deuterium occupying 27 108 sites forms 124 510 deuterium–metal correlations, and the ratio of each correlation to the total is shown for each occupied site (Fig. 7 ▸). The empirical rule estimates that the hole sizes are greater than 0.4 Å, but the current study indicates that they are longer than 0.3 Å. The average structural analyses of neutron diffraction measurements indicate that deuterium occupies the *A*2*B*4, *A*2*B*2, *AB*3 and *B*4 sites in the *AB*_5_ system, but the *B*4 site has the lowest occupancy (Percheron-Guégan *et al.*, 1980 ▸; Achard *et al.*, 1982 ▸; Du *et al.*, 2003 ▸; Nakamura *et al.*, 2004 ▸). Theoretical investigations have similarly claimed that hydrogen occupies the *A*2*B*4, *A*2*B*2 and *AB*3 sites, but not the *B*4 site (Zhang *et al.*, 2006 ▸; Chen *et al.*, 2007 ▸; Zhang *et al.*, 2008 ▸). In the current study, the deuterium occupancy (ratio of deuterium to metal, D/*M*) in the initial structure was 0.57 at *A*2*B*4 and 0.31 at *A*2*B*2, while the modeling resulted in D/*M* of 0.26 at *A*2*B*4, 0.32 at *A*2*B*2, 0.27 at *AB*3 and 0.03 at *B*4 (Table 1 ▸). However, the *A*2*B*4 site is composed of three short (*r* ≃ 1.7 Å, hole size ≃ 0.5 Å) and one unusually long (*r* ≃ 2.5 Å, hole size ≃ 1.3 Å) D–*B* correlations, as shown in Fig. 7 ▸. By excluding the longest correlation, the occupied polyhedron was regarded as bipyramidal *A*2*B*3 (Guénée *et al.*, 2003 ▸) and the space larger than 1.2 Å in the distribution shown in Fig. 7 ▸ disappeared, thus approaching a normal distribution (Fig. S8).

Although the current model was analyzed without assuming empirical rules or the symmetry of the crystal structure, the obtained atomic arrangements are consistent with those previously reported. In addition, the Al ratios of the polyhedra occupied by deuterium are all lower than the compositional average (Table 1 ▸). There is no bias in the occupancy and hole sizes of the *A*2*B*4, *A*2*B*2 and *AB*3 sites occupied by deuterium, and the compositions are lower than the Al average composition of 0.0948, suggesting that deuterium is avoiding Al for chemical reasons. Theoretical calculations indicate that hydrogen tends to avoid aluminium atoms and would not be stationed at those interstitial sites partly surrounded by Al (Zhang *et al.*, 2006 ▸). This is in good agreement with the experimental finding that the hydrogen content of hydrides is reduced with the substitution of Al for Ni (Percheron-Guégan *et al.*, 1985 ▸), which is also consistent with the trend observed in the current study. Average structural analysis using Rietveld or PDF refinements does not provide this information because they cannot distinguish between elements occupying the same site, and they only address small differences in atomic displacement. Local structural analysis, such as that carried out in the current study, provides essential information for the future development of hydrogen storage materials.

## 4. Conclusions

We developed two types of *in situ* gas-loading setups for two-dimensionally arranged detectors in time-of-flight neutron total scattering experiments for hydrogen storage materials. A chemically stable single-crystal sapphire container operated at 473 K and 10 MPa hydrogen gas pressure was used to detect the deuterium absorption process of palladium within a few seconds. The local structure of an La–Ni–Al alloy sealed in a double-walled vanadium container was also investigated using high-resolution real-space PDF data obtained by a neutron total scattering experiment and RMC structural modeling. The deuterium–deuterium shortest distance and deuterium occupancy hole size in LaNi_4.53_Al_0.47_D_5.27_ were determined experimentally. Avoiding Al, deuterium occupies interstitial sites, and the hole sizes were found to be greater than 0.3 Å, which is slightly less than the empirically determined value of 0.4 Å.

## Supplementary Material

Additional tables and figures. DOI: 10.1107/S1600576722010561/ei5089sup1.pdf

## Figures and Tables

**Table 1:** Deuterium to metal ratio (D/*M*) at occupation sites and Al composition ratio Al/(Ni+Al) of deuterium occupation sites for LaNi_4.53_Al_0.47_D_5.27_ (PDF refinement) and La_5131_Ni_23207_Al_2419_D_27108_ (RMC modeling)

	D/*M* ratio		Al composition ratio	
Refinement/modeling	PDF	RMC	PDF	RMC
*O* (*A*2*B*4)	0.5728	0.261	0.0948	0.0779
*T* (*A*2*B*2)	0.3050	0.323	0.0948	0.0240
*T* (*AB*3)	–	0.268	–	0.0382
*T* (*B*4)	–	0.029	–	0.0913

## References

[bb1] Achard, J. C., Dianoux, A. J., Lartigue, C., Percheron-Guégan, A. & Tasset, F. (1982). *Structure of Al, Cu and Si Substituted LaNi_5_ and of the Corresponding B-Deuterides from Powder Neutron Diffraction.* New York: Plenum.

[bb2] Chen, D., Gao, T., Li, G., Zhou, J. & Tang, L. (2007). *Solid State Commun.* **141**, 378–383.

[bb3] Crowder, C., James, W. J. & Yelon, W. (1982). *J. Appl. Phys.* **53**, 2637–2639.

[bb4] Du, H., Zhang, W., Wang, C., Han, J., Yang, Y., Chen, B., Xie, C., Sun, K. & Zhang, B. (2003). *Solid State Commun.* **128**, 157–161.

[bb5] Egami, T. & Billinge, S. J. L. (2003). *Underneath the Bragg Peaks.* San Diego: Elsevier.

[bb6] Farrow, C. L., Juhas, P., Liu, J. W., Bryndin, D., Božin, E. S., Bloch, J., Proffen, T. & Billinge, S. J. (2007). *J. Phys. Condens. Matter*, **19**, 335219. 10.1088/0953-8984/19/33/335219 21694142

[bb7] Finger, R., Hansen, T. C. & Kohlmann, H. (2021). *QuBS*, **5**, 22.

[bb8] Finger, R., Hansen, T. C. & Kohlmann, H. (2022). *J. Appl. Cryst.* **55**, 67–73. 10.1107/S1600576721012048 PMC8805162 35145356

[bb9] Finger, R., Kurtzemann, N., Hansen, T. C. & Kohlmann, H. (2021). *J. Appl. Cryst.* **54**, 839–846. 10.1107/S1600576721002685 PMC8202029 34188615

[bb10] Fischer, P., Furrer, A., Busch, G. & Schlapbach, L. (1977). *Helv. Phys. Acta*, **50**, 421–430.

[bb11] Flanagan, T. B. & Oates, W. A. (1991). *Annu. Rev. Mater. Sci.* **21**, 269–304.

[bb12] Gray, E. M., Blach, T. P., Pitt, M. P. & Cookson, D. J. (2011). *J. Alloys Compd.* **509**, 1630–1635.

[bb13] Gray, E. MacA., Smith, R. I. & Pitt, M. P. (2007). *J. Appl. Cryst.* **40**, 399–408.

[bb14] Guénée, L., Favre-Nicolin, V. & Yvon, K. (2003). *J. Alloys Compd.* **348**, 129–137.

[bb15] Hansen, T. C. & Kohlmann, H. (2014). *Z. Anorg. Allg. Chem.* **640**, 3044–3063.

[bb16] Ikeda, K., Fujisaki, F., Otomo, T., Ohshita, H., Honda, T., Kawamata, T., Arima, H., Sugiyama, K., Abe, H., Kim, H., Sakaki, K., Nakamura, Y., Machida, A., Sato, T., Takagi, S. & Orimo, S. (2021). *Appl. Sci.* **11**, 8349.

[bb17] Iwase, K., Mori, K., Hishinuma, Y., Hasegawa, Y., Iimura, S., Ishikawa, H., Kamoshida, T. & Ishigaki, T. (2011). *Int. J. Hydrogen Energy*, **36**, 3062–3066.

[bb18] Joubert, J. M., Černý, R., Latroche, M., Percheron-Guégan, A. & Schmitt, B. (2006). *Acta Mater.* **54**, 713–719.

[bb19] Joubert, J.-M., Paul-Boncour, V., Cuevas, F., Zhang, J. & Latroche, M. (2021). *J. Alloys Compd.* **862**, 158163.

[bb20] Kohlmann, H., Kurtzemann, N., Weihrich, R. & Hansen, T. (2009). *Z. Anorg. Allg. Chem.* **635**, 2399–2405.

[bb21] Lartigue, C., Le Bail, A. & Percheron-Guégan, A. (1987). *J. Less-Common Met.* **129**, 65–76.

[bb22] Latroche, M., Joubert, J. M., Percheron-Guégan, A. & Bourée-Vigneron, F. (2004). *J. Solid State Chem.* **177**, 1219–1229.

[bb23] Latroche, M., Percheron-Guégan, A., Chabre, Y., Poinsignon, C. & Pannetier, J. (1992). *J. Alloys Compd.* **189**, 59–65.

[bb24] Lin, H.-J., Li, H.-W., Shao, H., Lu, Y. & Asano, K. (2020). *Mater. Today Energy*, **17**, 100463.

[bb25] Machida, A., Higuchi, K., Katayama, Y., Sakaki, K., Kim, H. & Nakamura, Y. (2014). *Acta Cryst.* A**70**, C360.

[bb26] McGreevy, R. L. & Pusztai, L. (1988). *Mol. Simul.* **1**, 359–367.

[bb27] Mohammadshahi, S. S., Webb, T. A., Gray, E. M. & Webb, C. J. (2017). *Int. J. Hydrogen Energy*, **42**, 6793–6800.

[bb28] Nakamura, Y. & Akiba, E. (2000). *J. Alloys Compd.* **308**, 309–318.

[bb29] Nakamura, Y., Ishigaki, T., Kamiyama, T. & Akiba, E. (2004). *J. Alloys Compd.* **384**, 195–202.

[bb30] Nakamura, Y., Nakamura, J., Iwase, K. & Akiba, E. (2009). *Nucl. Instrum. Methods Phys. Res. A*, **600**, 297–300.

[bb31] Nield, V. M. & Keen, D. A. (2001). *Diffuse Neutron Scattering from Crystalline Materials.* New York: Oxford University Press Inc.

[bb32] Oishi, R., Yonemura, M., Nishimaki, Y., Torii, S., Hoshikawa, A., Ishigaki, T., Morishima, T., Mori, K. & Kamiyama, T. (2009). *Nucl. Instrum. Methods Phys. Res. A*, **600**, 94–96.

[bb33] Oishi-Tomiyasu, R. (2012). *Acta Cryst.* A**68**, 525–535. 10.1107/S0108767312024579 22893236

[bb34] Okumura, M., Ikado, A., Saito, Y., Matsushita, Y., Aoki, H. & Kawakami, Y. (2021). *J. Jpn. Inst. Energ.* **100**, 5–12.

[bb35] Percheron-Guégan, A., Lartigue, C. & Achard, J. (1985). *J. Less-Common Met.* **109**, 287–309.

[bb36] Percheron-Guégan, A., Lartigue, C., Achard, J., Germi, P. & Tasset, F. (1980). *J. Less-Common Met.* **74**, 1–12.

[bb37] Rao, B. K. & Jena, P. (1985). *Phys. Rev. B*, **31**, 6726–6730. 10.1103/physrevb.31.6726 9935555

[bb38] Remhof, A., Friedrichs, O., Buchter, F., Mauron, P., Kim, J. W., Oh, K. H., Buchsteiner, A., Wallacher, D. & Züttel, A. (2009). *J. Alloys Compd.* **484**, 654–659.

[bb39] Sakaki, K., Akiba, E., Mizuno, M., Araki, H. & Shirai, Y. (2009). *J. Alloys Compd.* **473**, 87–93.

[bb40] Sakaki, K., Kim, H., Machida, A., Watanuki, T., Katayama, Y. & Nakamura, Y. (2018). *J. Appl. Cryst.* **51**, 796–801.

[bb41] Sakaki, K., Terashita, N., Kim, H., Proffen, T., Majzoub, E. H., Tsunokake, S., Nakamura, Y. & Akiba, E. (2013). *Inorg. Chem.* **52**, 7010–7019. 10.1021/ic400528u 23724781

[bb42] Sato, T., Ikeda, K., Matsuo, M., Miwa, K., Otomo, T., Deledda, S., Hauback, B. C., Li, G., Takagi, S. & Orimo, S. (2017). *Int. J. Hydrogen Energy*, **42**, 22449–22453.

[bb43] Sato, T., Mochizuki, T., Ikeda, K., Honda, T., Otomo, T., Sagayama, H., Yang, H., Luo, W., Lombardo, L., Züttel, A., Takagi, S., Kono, T. & Orimo, S. I. (2020). *ACS Omega*, **5**, 31192–31198. 10.1021/acsomega.0c04535 PMC7726944 33324828

[bb44] Switendick, A. C. (1979). *Z. Phys. Chem.* **117**, 89–112.

[bb45] Thompson, P., Reilly, J. J., Corliss, L. M., Hastings, J. M. & Hempelmann, R. (1986). *J. Phys. F*, **16**, 675–685.

[bb46] Toby, B. H. & Von Dreele, R. B. (2013). *J. Appl. Cryst.* **46**, 544–549.

[bb47] Tucker, M. G., Keen, D. A., Dove, M. T., Goodwin, A. L. & Hui, Q. (2007). *J. Phys. Condens. Matter*, **19**, 335218. 10.1088/0953-8984/19/33/335218 21694141

[bb48] Westlake, D. G. (1983*a*). *J. Less-Common Met.* **91**, 1–20.

[bb49] Westlake, D. G. (1983*b*). *J. Less-Common Met.* **91**, 275–292.

[bb50] Widenmeyer, M., Niewa, R., Hansen, T. C. & Kohlmann, H. (2013). *Z. Anorg. Allg. Chem.* **639**, 285–295.

[bb51] Yartys, V. A., Burnasheva, V. V., Semenenko, K. N., Fadeeva, N. V. & Solovev, S. P. (1982). *Int. J. Hydrogen Energy*, **7**, 957–965.

[bb52] Zhang, C. Y., Gao, T., Li, G. X., Zhang, Y. G. & Tang, L. J. (2008). *Solid State Commun.* **147**, 317–322.

[bb53] Zhang, R. J., Wang, Y. M., Chen, D. M., Yang, R. & Yang, K. (2006). *Acta Mater.* **54**, 465–472.

[bb54] Zhang, X., Sun, Y., Xia, G. & Yu, X. (2022). *J. Alloys Compd.* **899**, 163254.

