# Supplementary material for: Pressure cells for in situ neutron total scattering: time and real-space resolution during deuterium absorption
Source: J Appl Crystallogr. 2022 Dec 1;55(Pt 6):1631–9. doi: 10.1107/S1600576722010561 (PMC9721332; doi:10.1107/S1600576722010561)
Supplement: Supplementary file 1 [file j-55-01631-sup1.pdf]

JOURNAL OF
APPLIED
CRYSTALLOGRAPHY

Volume 55 (2022)

Supporting information for article:

Pressure cells for *in situ* neutron total scattering: time and real-space resolution during deuterium absorption

Kazutaka Ikeda, Hidetoshi Ohshita, Toshiya Otomo, Kouji Sakaki, Hyunjeong Kim, Yumiko Nakamura, Akihiko Machida and Robert B. Von Dreele

Table S1 Results of Rietveld and PDF refinements with space group $Fd\bar{3}m$ (no. 227) of Si (NIST SRM 640d).

Refinement		Rietveld			PDF		
Container		Single-crystal sapphire	In situ vanadium	Ex situ vanadium	Single-crystal sapphire	In situ vanadium	Ex situ vanadium
Fit range	*d* or *r*/Å	0.6–4.0	0.4–4.0	0.4–4.0	0.5–20.0	0.5–20.0	0.5–20.0
Unit cell	*a*/Å	5.43123	5.43123	5.43123	5.4225(2)	5.420440(13)	5.423610(8)
Si	U/Å^2	0.00718(6)	0.00491(2)	0.00525(17)	0.0308(8)	0.006745(17)	0.006820(11)
Gaussian fit of 111	FWHM/Å	0.1805(7)	0.1806(7)	0.1802(7)	-	-	-
Reliability	R_{wp}/%	1.79	3.48	4.33	12.9	13.0	12.0

U: isotropic atomic displacement, R_{wp}: weighted reliability. Standard deviations are in parentheses and refer to the last digit. Parameters without standard deviations were fixed in the refinement.

Table S2 Results of Rietveld and PDF refinements with space group *P*6/*mmm* (no. 191) of $LaNi_{4.5}Al_{0.5}$ before and after deuterium absorption at room temperature.

Compound		Alloy		Hydride			
Refinement		Rietveld	PDF	Rietveld	PDF	Rietveld[a]	Rietveld[b]
Unit cell	*a*/Å	5.042911(9)	5.04210(2)	5.354025(15)	5.35283(5)	5.313(1)	5.353(1)
	c/Å	4.027995(11)	4.02472(4)	4.274487(18)	4.27084(7)	4.242(1)	4.263(1)
	V/Å^3	88.71196	88.8513	106.1147	106.442	103.7	105.8
La	*U*/Å^2	0.00613(6)	0.013(1)	0.00786(7)	0.014(1)	0.0139(9)	0.0171(9)
1*a* (0, 0, 0)	*g*	1	1	1	1	0.99	0.99
Ni1	*U*/Å^2	0.00438(4)	0.013(1)	0.01545(4)	0.021(1)	0.0209(6)	0.208(7)
2*c* (1/3, 2/3, 0)	*g*	1	1	1	1	0.970	1
Ni2	*U*/Å^2	0.00438(4)	0.010(1)	0.01545(4)	0.016(1)	0.0128(3)	0.0189(4)
3*g* (1/2, 0, 1/2)	*g*	0.8680(4)	0.8420(8)	0.8814(6)	0.8420	0.687	0.860
Al2	*U*/Å^2	0.00438(4)	0.010(1)	0.01545(4)	0.016(1)	0.0128(3)	0.0189(4)
3*g* (1/2, 0, 1/2)	*g*	0.1320(4)	0.1580(8)	0.1186(6)	0.1580	0.313	0.140
D1	*z*	-	-	0.11996(6)	0.10068(7)	0.103[b]	0.103[d]
6*i* (1/2, 0, *z*)	*U*/Å^2	-	-	0.03771(15)	0.022(1)	0.0456(2)[b]	0.0272(12
O [A2B4]	*g*	-	-	0.4848(5)	0.5728(8)	0.456[b]	0.436[d]
D2	*x*	-	-	0.14625(5)	0.13756(5)	0.137	0.138
6*m* (*x*, 2*x*, 1/2)	*U*/Å^2	-	-	0.03771(15)	0.048(1)	0.060(4)	0.0272(12
T [A2B2]	*g*	-	-	0.3466(5)	0.3050(6)	0.343	0.318
D3	*z*	-	-	-	-	-	0.398
4*h* (1/3, 2/3, *z*)	*U*/Å^2	-	-	-	-	-	0.0272(12
T [AB3]	*g*	-	-	-	-	-	0.058
D4	*x*	-	-	-	-	-	0.225
12*o* (*x*, 2*x*, *z*)	*z*	-	-	-	-	-	0.319
T [B4]	*U*/Å^2	-	-	-	-	-	0.0272(12
	g	-	-	-	-	-	0.053
Reliability	R_{wp}/%	3.29	6.46	5.08	12.85	7.11	4.94
Composition		$LaNi_{4.60}Al_{0.40}$	$LaNi_{4.53}Al_{0.47}$	$LaNi_{4.64}Al_{0.36}D_{4.99}$	$LaNi_{4.53}Al_{0.47}D_{5.27}$	$LaNi_{4.12}Al_{0.95}D_{4.8}$	$LaNi_{4.64}Al$

g: Occupancy, *U*: isotropic atomic displacement, R_{wp}: weighted reliability. Standard deviations are in parentheses and refer to the last digit. Parameters without standard deviations were fixed in the refinement.

[a] Percheron-Guégan *et al.*, 1980.

[b] Achard *et al.*, 1982.

[c] Converted values from the 12*n* (0.471(3), 0, 0.103(1)) site.

[d] Converted values from the 12*n* (0.470(2), 0, 0.103(1)) site.

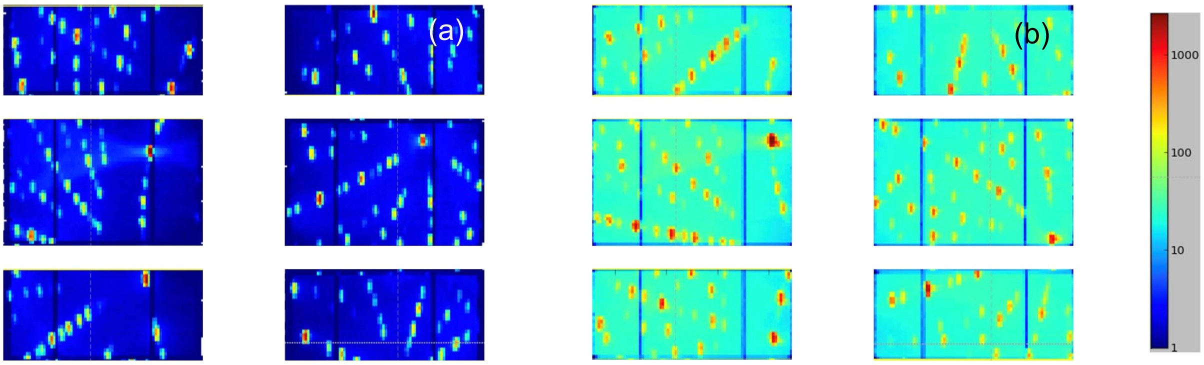

Figure S1 Laue patterns detected at the left and right sides of the 90° detector bank for *d* = 0.5–3.5 Å of (a) the empty single-crystal sapphire container and (b) the container sealing Si powder.

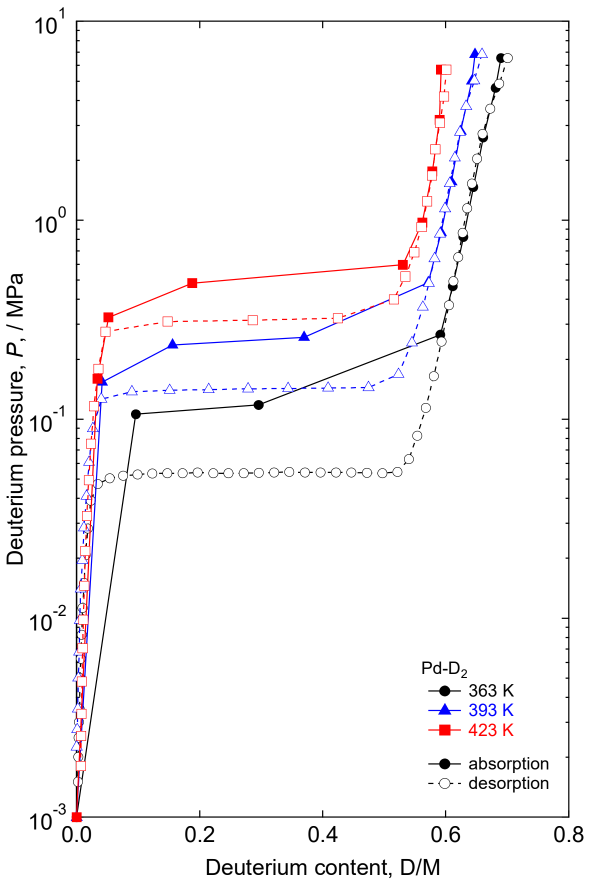

Figure S2 Hydrogen (deuterium) pressure–composition (*p*–*c*) isotherms at 363, 393, and 423 K for palladium powder.

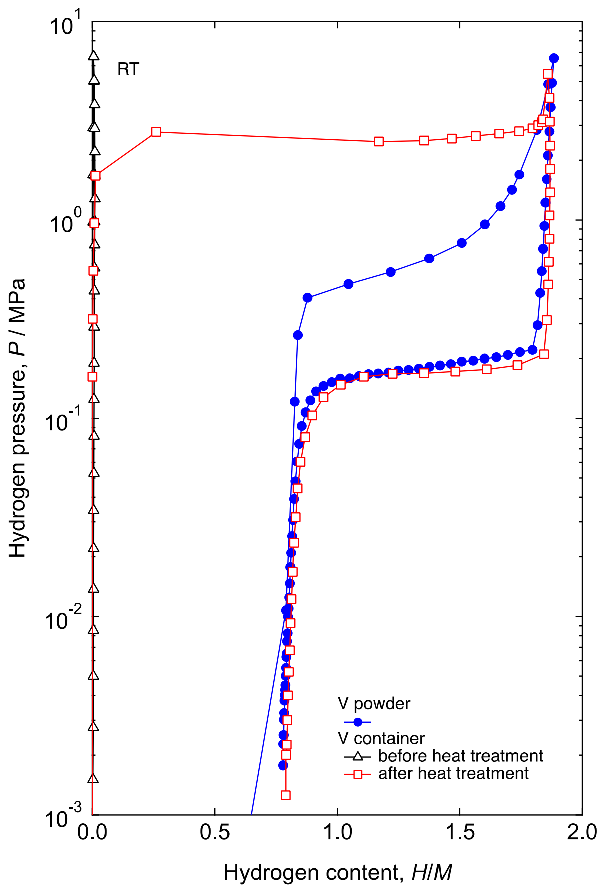

Figure S3 Hydrogen pressure–composition (*p*–*c*) isotherms at room temperature for vanadium powder and vanadium container before and after heat treatment at 773 K.

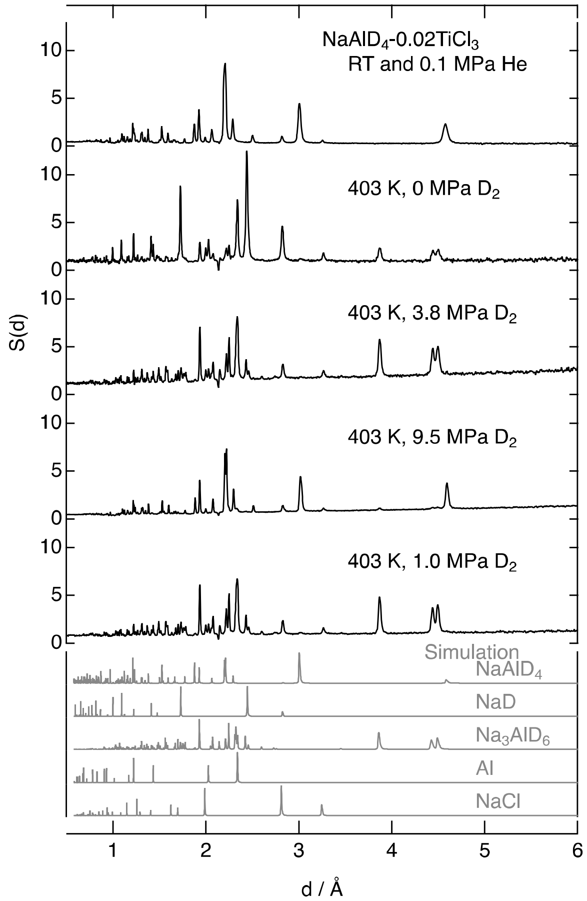

Figure S4 Neutron diffraction patterns of $NaAlD_4$–$0.02TiCl_3$ powder sealed in the in situ vanadium container.

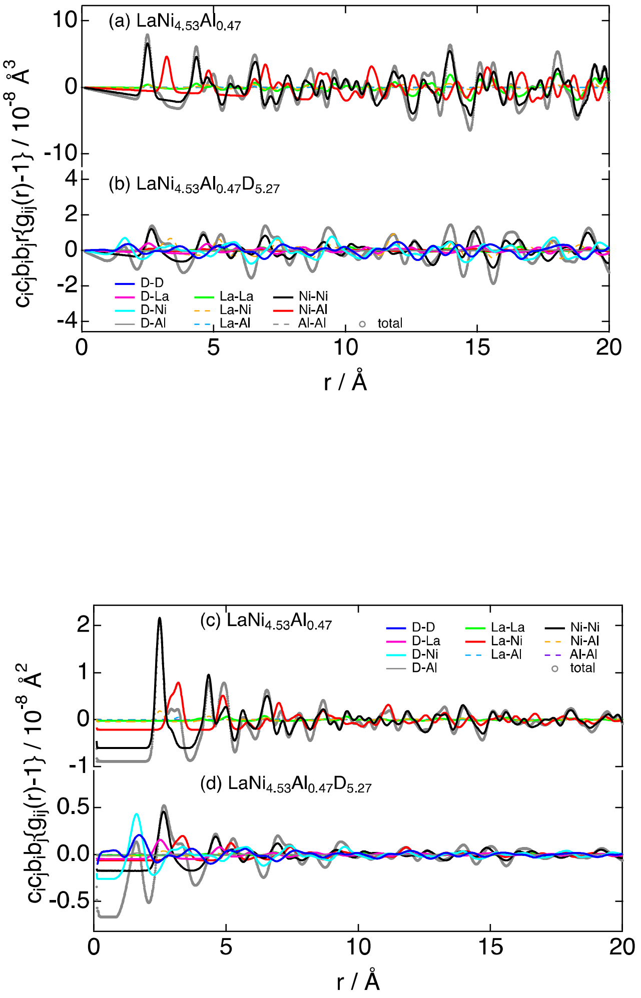

Figure S5 Two types of simulated neutron partial PDF patterns ($c_i c_j b_i b_j r\{g_{ij}(r) - 1\}$ and $c_i c_j b_i b_j \{g_{ij}(r) - 1\}$) of $LaNi_{4.5}Al_{0.5}$ (a) and (c) before and (b) and (d) after deuterium absorption at room temperature.

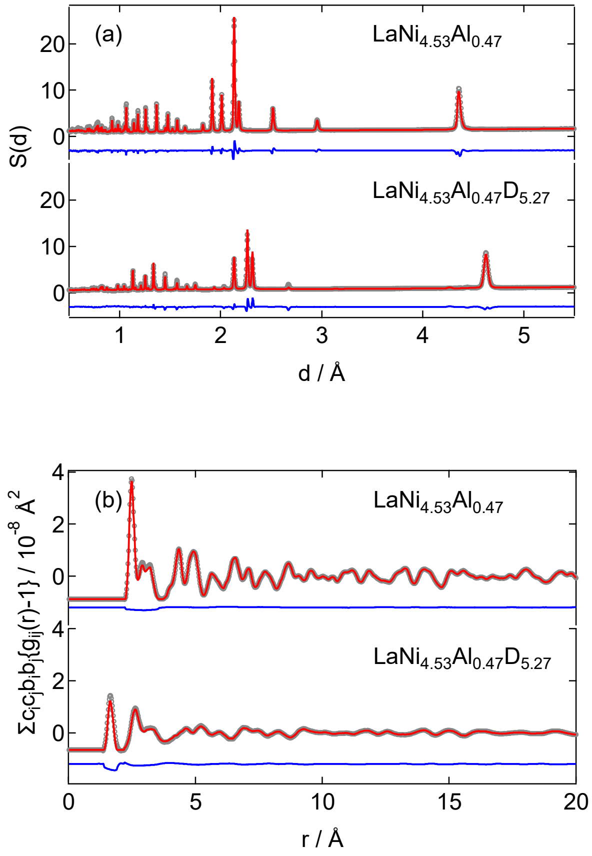

Figure S6 RMC modeling results for (a) Bragg scattering and (b) $g(r)$ of $LaNi_{4.5}Al_{0.5}$ before and after deuterium absorption at room temperature.

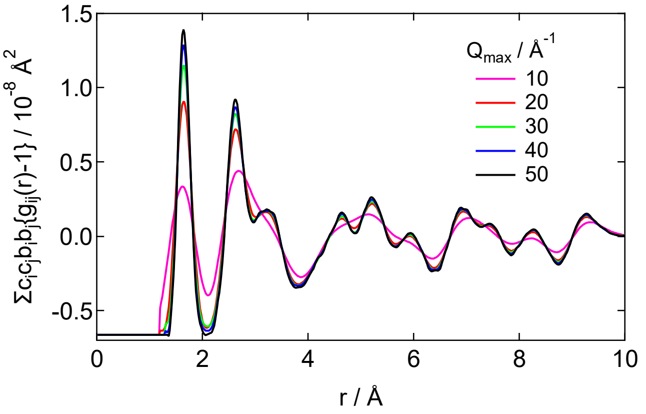

Figure S7 $Q_{\max}$ dependence of the Fourier transform to obtain the pair distribution function of $LaNi_{4.53}Al_{0.47}D_{5.27}$. To separate the first (D–Ni/Al) and second (D–La, Ni–Ni, and Ni–Al) pair correlation peaks, $Q_{\max}$ = 50 Å^{-1} was needed to make $g(r) = 0$ between the peaks.

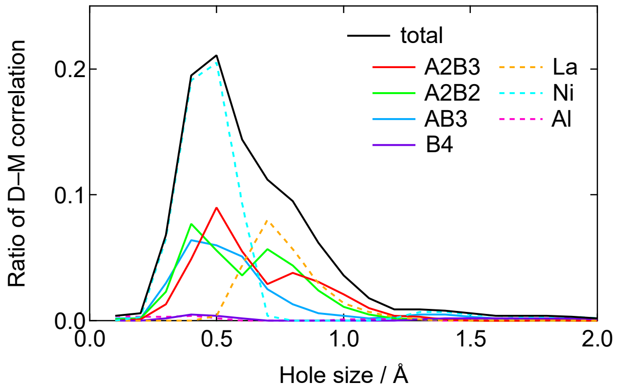

Figure S8 Hole-size distribution of deuterium occupation sites of A2B3, A2B2, AB3, and B4 for $LaNi_{4.5}Al_{0.5}$ after deuterium absorption, indicated using the ratio of each D–M correlation to the total. The distribution is also shown using the ratio to constituent elements.
